# Advancing Laboratory Diagnostics for Future Pandemics: Challenges and Innovations

**DOI:** 10.3390/pathogens14111135

**Published:** 2025-11-09

**Authors:** Lechuang Chen, Qing H. Meng

**Affiliations:** Department of Laboratory Medicine, The University of Texas MD Anderson Cancer Center, 1515 Holcombe Blvd., Unit 37, Houston, TX 77030, USA

**Keywords:** epidemic, pandemics, SARS, H1N1pdm09, Ebola, COVID-19, diagnostic technologies

## Abstract

Since the beginning of the 21st century, major epidemics and pandemics such as SARS, H1N1pdm09, Ebola, and COVID-19 have repeatedly challenged global systems of disease diagnostics and control. These crises exposed the weaknesses of traditional diagnostic models, including long turnaround times, uneven resource distribution, and supply chain bottlenecks. As a result, there is an urgent need for more advanced diagnostic technologies and integrated diagnostics strategies. Our review summarizes key lessons learned from four recent major outbreaks and highlights advances in diagnostic technologies. Among these, molecular techniques such as loop-mediated isothermal amplification (LAMP), transcription-mediated amplification (TMA), recombinase polymerase amplification (RPA), and droplet digital polymerase chain reaction (ddPCR) have demonstrated significant advantages and are increasingly becoming core components of the detection framework. Antigen testing plays a critical role in rapid screening, particularly in settings such as schools, workplaces, and communities. Serological assays provide unique value for retrospective outbreak analysis and assessing population immunity. Next-generation sequencing (NGS) has become a powerful tool for identifying novel pathogens and monitoring viral mutations. Furthermore, point-of-care testing (POCT), enhanced by miniaturization, biosensing, and artificial intelligence (AI), has extended diagnostic capacity to the front lines of epidemic control. In summary, the future of epidemic and pandemic response will not depend on a single technology, but rather on a multi-layered and complementary system. By combining laboratory diagnostics, distributed screening, and real-time monitoring, this system will form a global diagnostic network capable of rapid response, ensuring preparedness for the next global health crisis.

## 1. Introduction

Emerging and re-emerging infectious diseases are among the most serious challenges to global public health [1]. Since the beginning of the 21st century, outbreaks including severe acute respiratory syndrome (SARS), the 2009 influenza virus H1N1 (H1N1pdm09) pandemic, the West African Ebola epidemic, and the Coronavirus Disease 2019 (COVID-19) pandemic have repeatedly tested the world’s response capacity. Each of these events has also driven major advances in infectious disease diagnostics and control technologies. Accurate, efficient, and accessible diagnostic tools are essential for outbreak surveillance, case identification, interruption of transmission chains, and the design of control strategies. However, traditional diagnostic models that depend on centralized laboratories often face obstacles during the early stages of epidemics, including long turnaround times, unequal distribution of resources, and fragile supply chains [2]. Developing and integrating new diagnostic technologies to create a rapid, globally responsive epidemic and pandemic diagnostic network has therefore become a critical task in the post-pandemic era.

Lessons from past epidemics continue to guide our strategy. During the 2003 SARS outbreak, it was difficult in the early stages to distinguish SARS from other respiratory infections, delaying timely control [3]. The 2009 H1N1pdm09 pandemic revealed critical gaps in global surveillance infrastructure [4]. The 2014–2016 Ebola epidemic highlighted the value of fast turnaround times and revealed how mobile diagnostic laboratories could be decisive during outbreaks [5]. The COVID-19 pandemic accelerated diagnostic innovation but also revealed severe supply chain disruptions and major gaps in global diagnostic capacity [6]. It further underscores the need for international cooperation: an epidemic left unchecked in one country or region can quickly grow into a worldwide health crisis [7].

In the past two decades, diagnostic technology has advanced rapidly, transforming our ability to detect and characterize pathogens. Molecular diagnostics, particularly nucleic acid amplification tests (NAATs), are considered the “gold standard” for pathogen detection due to their high sensitivity and specificity [8]. However, the COVID-19 pandemic revealed the limits of conventional PCR, which requires complex laboratory instruments, has longer turnaround times, and is easily affected by supply shortages [2]. These challenges led to the use of newer molecular technologies such as LAMP [9], TMA [10], RPA [11], and ddPCR [12]. Antigen testing has been shown to be an important complement, offering fast results (typically within 15–30 min), ease of use, and lower costs, and is particularly valuable for community screening [13]. Serological testing, by detecting antibodies such as IgM and IgG, provides unique value for retrospective outbreak analysis and assessment of population immunity [14]. NGS, once limited to research, is becoming an important feature of public health systems, which can detect new pathogens, variants, and critical mutations [15]. In addition, POCT devices, which deliver results within minutes, bring diagnostic capabilities closer to the bedside and support rapid clinical decision-making [16]. In summary, epidemic diagnostics can no longer rely on single technology or a centralized laboratory. Instead, they require a multi-layered and complementary system that integrates molecular diagnostics, antigen testing, serological monitoring, genomic sequencing, and point-of-care testing. We review the experiences and lessons from four major epidemics, discuss the principles, advantages, applications, and progress of key diagnostic approaches, and consider strategies for building a more flexible and responsive global diagnostic network to address future public health crises.

## 2. Search Methodology

To ensure a comprehensive and relevant analysis for this review, a systematic literature search was conducted. The primary objective was to identify key scientific publications, reports, and guidelines concerning laboratory diagnostics during the major epidemics and pandemics of the 21st century (specifically SARS, Ebola, H1N1pdm09, and COVID-19) and emerging diagnostic technologies.

### 2.1. Information Sources and Search Strategy

Searches were performed in the databases PubMed/MEDLINE and Web of Science. The search strategy combined keywords and Medical Subject Headings (MeSH) terms related to three core concepts: (1) the specific outbreaks (e.g., “SARS”, “H1N1pdm09”, “Ebola”, “COVID-19”, “epidemic”, “pandemic”); (2) diagnostic techniques (e.g., “molecular diagnostics”, “PCR”, “antigen test”, “serology”, “next-generation sequencing”, “point-of-care testing”, “infectious virus quantification”); and (3) broader public health context (e.g., “diagnostic capacity”, “turnaround times”, “surveillance”, “lessons learned”).

Operators (and, or) were used to combine these terms. For example, a search string was structured as: (“COVID-19” or “pandemic”) and (“antigen detection” or “rapid diagnostic test”) and (“sensitivity” or “public health”).

### 2.2. Eligibility Criteria

The literature screening and selection were based on the following criteria:

Inclusion Criteria: Peer-reviewed original research articles, review articles, systematic reviews, and meta-analyses. Official reports and guidelines from major international health organizations, such as World Health Organization (WHO), U.S. Centers for Disease Control and Prevention (CDC), European Centre for Disease Prevention and Control (ECDC) were also included. The timeframe for considered publications was primarily from 2000 to 2025 to cover the relevant outbreaks and technological advances.

Exclusion Criteria: Non-English publications, conference abstracts without full text, and articles not directly relevant to diagnostic strategies or lessons from the specified public health emergencies were excluded.

### 2.3. Study Selection

The initial database searches yielded a large volume of records. The selection process involved a two-stage screening. First, titles and abstracts were screened for relevance to the review’s themes. Subsequently, the full texts of potentially relevant articles were retrieved and assessed for eligibility based on the inclusion and exclusion criteria. Furthermore, a manual search of the reference lists of key review articles was performed to identify additional pertinent publications that may not have been captured by the electronic database search. This process ensured the inclusion of seminal and high-impact literature that forms the evidence base for the discussions and conclusions presented in this review.

## 3. Lessons Learned from Past Epidemics and Pandemics

### 3.1. Severe Acute Respiratory Syndrome (SARS) Outbreak (2002–2003)

The SARS outbreak began in November 2002 and rapidly spread from China to neighboring countries such as Vietnam and Singapore, ultimately affecting 29 countries and regions worldwide [17]. In the early stages, diagnostics and control were severely challenged by the unknown source of infection and the lack of a confirmed viral identity. According to the WHO, as late as April 2003, the three available diagnostic methods, including PCR testing, immunofluorescence assay (IFA), and enzyme-linked immunosorbent assay (ELISA), were still of limited effectiveness [18]. Clinicians often had to rely on patients’ travel history, hospitalization records, and contact tracing for diagnosis.

This outbreak demonstrated that laboratory diagnosis was especially difficult during early transmission. Although patients were most infectious in the first week, viral loads in respiratory and stool samples were typically low during this period, reducing the sensitivity of PCR. Nevertheless, PCR was highly specific and able to detect as few as 1–10 copies of viral RNA [3]. Serological testing performed somewhat better, identifying SARS coronavirus RNA in more than 50% of patients within the first week, but this still fell short of clinical needs [19]. The SARS epidemic also exposed weaknesses in epidemic diagnostics systems, including incomplete laboratory infrastructure, poor coordination between departments, and delays in releasing testing and infection control guidelines. These weaknesses slowed both virus identification and transmission control. Surveillance systems in many countries were not prepared for an outbreak of such scale. For example, the UK Health Protection Agency initially classified the risk as “low,” but later had to manage as many as 368 suspected SARS cases in a few months [20]. The systems for tracking cases and viruses could not keep up with the outbreak. Local staff often did not have the power to record or manage case reports and contact details. This led to delays in data sharing [21].

Despite these challenges, the outbreak led to major investments in diagnostic and monitoring systems, especially in Asia. It highlighted the urgent need to improve diagnostic technologies and diagnostics strategies. It also pointed out the importance of having up-to-date national databases for tracking cases and laboratory results. Most importantly, SARS reminded the world that viruses spread across countries and require global cooperation [22]. The lessons learned from SARS directly informed many countries’ early response strategies during the COVID-19 pandemic.

### 3.2. H1N1pdm09 Pandemic (2009–2010)

The 2009 H1N1pdm09 pandemic, which was first reported in North America, April 2009. It was the first influenza pandemic of the 21st century and provided a critical stress test for global influenza surveillance systems. This pandemic ultimately affected over 214 countries and territories, approximately 60.8 million cases, 274,000 hospitalizations, and 12,469 deaths in the United States alone during the first year. Unlike seasonal influenza, H1N1pdm09 pandemic significantly affected younger populations, with 87% of deaths occurring in people under 65 years of age [23]. This pandemic presented unprecedented challenges to clinical laboratories worldwide. In the United States, a survey of 931 laboratories revealed that the number of centers using NAATs more than doubled during the outbreak. This H1N1pdm09 pandemic accelerated adoption of RT-PCR as the primary diagnostic method for influenza detection. All national laboratories supported through capacity-strengthening programs implemented RT-PCR technology, with diagnostic accuracy improving dramatically. And laboratories recognized the limitations of rapid antigen tests, which demonstrated sensitivity of only 40–69% for H1N1pdm09 virus detection [24].

This pandemic exposed critical gaps in global influenza surveillance capacity. At the time of the outbreak, 106 (54%) of 193 WHO Member States had no or very limited seasonal influenza surveillance systems [4]. By July 2009, as laboratory-confirmed cases approached 100,000 globally, it became evident that case-based surveillance was overwhelming national laboratory systems. WHO subsequently issued new surveillance guidelines, shifting focus from universal testing to targeted testing of severe cases and fatal outcomes [4]. The H1N1pdm09 pandemic led to fundamental changes to global influenza surveillance infrastructure. The WHO Global Influenza Surveillance and Response System (GISRS) was significantly strengthened, with substantial increases in both laboratory capacity and sentinel surveillance sites. Between 2004–2013, the number of countries conducting routine virologic surveillance increased from 19 to 35, while annual specimen testing grew from 81,851 to 542,235 specimens [25]. Importantly, 80% of countries reported leveraging the enhanced influenza surveillance platforms developed during this period to detect other respiratory pathogens. This multi-pathogen surveillance capability proved invaluable during subsequent outbreaks, including the COVID-19 pandemic [25].

This pandemic also led to revisions to global pandemic preparedness frameworks. The CDC updated its preparedness framework from the previous stage-based approach to a more detailed six-interval system with eight response domains, providing greater clarity for decision-making during future pandemics [26]. Similarly, the ECDC and Control developed enhanced guidance for pandemic preparedness plan revision, incorporating lessons learned from H1N1pdm09 pandemic [27]. The establishment of the WHO FluID (Flu Informed Decisions) platform for direct epidemiological data reporting and the enhancement of existing surveillance networks like FluNet represented direct responses to coordination challenges identified during the pandemic. These systems created standardized reporting mechanisms that facilitated more effective international surveillance coordination [28].

### 3.3. West African Ebola Outbreak (2014–2016)

The West African Ebola outbreak, which began in December 2013, became the largest and most severe Ebola virus disease epidemic in history [29]. After 28 months, the outbreak resulted in 28,652 cases and 11,325 deaths [30]. A central challenge was the lack of local diagnostic capacity in affected countries such as Guinea, Liberia, and Sierra Leone. Most samples had to be shipped to international reference laboratories, a process that often required several days or even weeks [31]. For example, at the peak of the epidemic in Sierra Leone (October–November 2014), limited laboratory instruments and poor transport conditions led to result reporting delays of more than two days, and sometimes up to a week. During this time, patients crowded into holding centers, and contacts were not traced quickly. This allowed the virus to spread without control [32]. Although PCR testing was very accurate, it was hard to keep running in local conditions. Frequent power outages and hot weather made cold-chain maintenance nearly impossible. Lack of training and insufficient protective equipment for staff also raised the risk of infection during blood collection. In addition, the cost, about $100 per test, was far more than local budgets could handle. All these barriers made Ebola much harder to quickly control the outbreak in West Africa [33]. The introduction of mobile diagnostic laboratories transformed the situation in some regions. For example, mobile labs from organizations like MRIGlobal reduced sample turnaround time from over two days to less than four hours and processed up to 110 samples daily [34]. Similarly, in Sierra Leone, Public Health England operated the GeneXpert platforms and delivered results in about 2.5 h. It demonstrated excellent agreement with the laboratory’s RT-PCR [35]. In Liberia, staff members were trained and deployed ten GeneXpert platforms, and instituting shift work increased testing volume eightfold, cleared backlogs, and reduced turnaround time to under 24 h. These steps made it possible to find and control two later Ebola clusters in time [36].

This outbreak highlighted several important lessons. Testing must be available at the frontlines and spread across different locations. One central lab, no matter how advanced, cannot meet the fast needs of remote areas. It also underscored the resource challenges faced by low- and middle-income countries (LMICs), where about 84% of the world’s population resides [37]. Effective global health assistance must go beyond supplying equipment, also need to ensure this equipment is functional, sustainable, and accessible in LMICs. A critical barrier is the operating cost beyond the initial procurement. This includes recurring expenses for reagents, quality control materials, and the often-overlooked spare parts and maintenance for sophisticated equipment. Studies have shown that the annual cost of spare parts can range from 0.25% for simple devices to 10% for high-technology medical devices of their total acquisition cost [38]. Frequent equipment downtime due to lack of technical support, spare parts, or unstable energy supply remains a major impediment to consistent diagnostic services [39].

Building diagnostic systems in LMICs requires strategies tailored to regional contexts. In Africa, integrated diagnostic models are being explored to optimize resources and infrastructure. These models, which include facility-based “one-stop shops” and the use of multi-disease testing platforms, aim to leverage existing health programs (e.g., HIV or maternal health services) to provide diagnostics for a broader set of conditions, such as tuberculosis, hypertension, and diabetes [40]. In Latin America, there is a growing movement to bolster regional diagnostic manufacturing to enhance supply chain resilience and reduce dependency on imports. Countries like Brazil, Argentina, and Cuba are strengthening their manufacturing bases, with a focus on developing diagnostics for regionally relevant diseases like dengue, chikungunya, and Zika. Initiatives such as the Pan American Health Organization’s (PAHO) Special Program, Innovation and Regional Production Platform are promoting regional cooperation to create a more self-reliant and sustainable health technology landscape [41].

Ultimately, lasting epidemic response demands a shift from only donating equipment to investing in entire health systems. This includes building local technical capacity for maintenance and repair, securing sustainable financing for operational costs, strengthening supply chains for reagents and spare parts, ensuring stable energy infrastructure, and teamwork across different departments. Without these parts, external aid cannot be fully translated into local diagnostic capacity [42].

### 3.4. Coronavirus Disease (2019–2023)

The COVID-19 pandemic, which began at the end of 2019, spread rapidly to more than 199 countries and regions by March 2020 [43]. By 31 December 2023, countries had reported about 772 million confirmed cases and over 7.0 million deaths to the WHO [44]. According to a WHO report released in 2024, the pandemic caused a decline of 1.8 years in global average life expectancy between 2019 and 2021, the sharpest drop in recent decades [45].

From the earliest stages, the pandemic pushed forward innovation in diagnostics. By April 2020, more than 590 different COVID-19 diagnostic tests had already been developed worldwide, and this trend continued in subsequent years [46]. Conventional PCR testing achieved large-scale deployment never seen before, by mid-June 2021, the United States alone had performed over 457 million SARS-CoV-2 tests [47]. This large testing capacity was made possible by automated high-volume machines and diverse testing strategies. At the same time, rapid antigen tests (RATs) were widely used in schools, workplaces, and communities. These tests made it possible to quickly find highly infectious individuals and breaking transmission chains [48]. The pandemic also highlighted the limitations of certain diagnostic methods. The accuracy of nucleic acid testing varies with sample type, collection timing, and quality. For example, lower respiratory tract samples like bronchoalveolar lavage (BAL) fluid, often collected from patients with severe pneumonia, demonstrated a high positivity rate (93%) in early studies, reflecting the pulmonary tropism of SARS-CoV-2. In contrast, upper respiratory samples like throat swabs showed lower sensitivity (32% in the same study), particularly outside the peak viral shedding period [49]. While saliva and nasopharyngeal swabs are more practical for large-scale testing, their sensitivity is also time-dependent, generally being highest around the time of symptom onset. Similarly, the “window period” of serological testing reduced its value in early diagnosis, as IgM and IgG antibodies generally became detectable only 3–14 days after symptom onset [50]. These findings confirmed that no single diagnostic technology is sufficient, and that selection must fit the clinical scenario [51]. Another major challenge revealed by COVID-19 was global supply chain fragility. Disruptions in the supply of medical materials and cold-chain transport caused widespread failures when testing needs rose quickly. The “just-in-time” supply model collapsed under the huge growth in demand. This demonstrated the need for backup reserves of key reagents and supplies, as well as diversified supply channels, to prepare for future outbreaks [52]. The pandemic underscored that effective epidemic response requires a multi-level and complementary diagnostic system (Table 1). Future epidemic diagnostics systems must integrate these platforms into a coordinated diagnostic network, while ensuring consistent quality across testing sites [53].

## 4. Technological Advances in Epidemic and Pandemic Diagnostics

### 4.1. Molecular Diagnostics

Molecular diagnostics, particularly NAATs, have long been regarded as the gold standard for pathogen detection [54]. Their high sensitivity and specificity make them essential for confirming infections, guiding patient isolation, and monitoring community transmission. However, the pandemic exposed limitations of conventional PCR testing and led to rapid development and application of alternative NAATs such as LAMP, TMA, RPA, and ddPCR.

LAMP has gained attention because it operates at a constant temperature (60–65 °C) without requiring an expensive thermal cycler. Compared with conventional PCR, it offers faster processing, fewer requirements for sample purification, and better adaptability in resource-limited settings [9]. Advanced platforms such as SMART-LAMP can process up to 40,000 samples per day while maintaining sensitivity comparable to conventional PCR, making it valuable for large-scale community screening [55]. TMA uses reverse transcriptase and RNA polymerase to amplify nucleic acids (often rRNA) at a constant temperature of about 42 °C. Its extremely high sensitivity makes it particularly suited for pathogen screening, and it is already widely used in blood screening [10]. Unlike LAMP, TMA systems are commonly integrated into high-throughput, fully automated platforms that provide closed workflows from sample handling to result analysis. RPA operates at even lower temperatures (37–42 °C). Recombinase-primer complexes invade double-stranded DNA to locate homologous sequences, and amplification proceeds with the help of single-stranded DNA binding proteins and strand-displacing DNA polymerases. Because the temperature requirements are minimal, the reaction can theoretically be driven by body heat. This flexibility makes RPA a strong candidate for true point-of-care testing (POCT). It also produces results quickly (10–20 min), though the technology is still maturing compared with LAMP and TMA [11]. ddPCR represents a major advance in quantitative diagnostics. Unlike quantitative real-time reverse transcription PCR, it partitions reactions into tens of thousands of discrete, water-in-oil droplets, allowing direct counting of positives and absolute quantification of viral nucleic acids without standard curves. It shows greater tolerance to inhibitors and is especially useful for detecting low viral loads and monitoring disease progression [12]. For example, in immunocompromised patients with persistent COVID-19, ddPCR has proven critical for detecting and quantifying sub-genomic RNA, a marker of viral replication, at levels often undetectable by conventional PCR [56]. With analysis efficiency of up to 83% and a coefficient of variation as low as 2%, ddPCR delivers high precision [57]. In addition, emerging platforms such as nanotechnology, improved both sensitivity and turnaround time [58]. Advances in multiplex PCR have further enabled simultaneous detection of dozens of respiratory pathogens, including SARS-CoV-2, influenza viruses, and respiratory syncytial virus, expanding both efficiency and breadth of pathogen surveillance [59]. This is particularly valuable in clinical settings during seasonal respiratory virus activity. A multiplex PCR panel can rapidly differentiate between SARS-CoV-2 and influenza in a patient presenting with febrile respiratory illness, directly informing the choice of antiviral therapy and infection control measures [60].

Another major direction in diagnostics is full automation combined with data cloud platform. For example, the new full automated PCR system performs every step, from sample storage and nucleic acid extraction to amplification and result analysis, without human intervention, and can run continuously for 24 h. This minimizes error and increases efficiency, even for diverse sample types such as blood, sputum, and feces [61]. Coupled with big data platforms, the system enables real-time diagnostic data collection and regional infection trend analysis, directly supporting clinical and public health decision-making.

### 4.2. Antigen Detection

Alongside NAATs, antigen tests were essential during the COVID-19 pandemic for quick sorting of patients and breaking transmission chains. These immunoassays detect viral surface proteins (antigens) and have clear advantages: they are simple to use, deliver results within 15–30 min, lower cost than NAATs, and can be widely used. These features made antigen tests particularly valuable for on-site screening in schools, workplaces, and communities [13]. The main limitation of antigen testing is its lower sensitivity compared with NAATs, as higher viral loads are typically required [62]. Evidence from the pandemic confirmed that antigen testing is most accurate during periods of peak viral load but may fail to detect infections in early or recovery stages [63].

To address these challenges, technical advances have focused on two areas: First, detection targets. Multi-target assays that detect both nucleocapsid and spike proteins reduce the risk of false negatives due to viral mutations [64]. Second, result interpretation. Smartphone-based readers and AI-driven algorithms have been developed to standardize interpretation and reduce subjective errors. Some advanced platforms can achieve sensitivity exceeding 95% in individuals with high viral loads, typically corresponding to a PCR cycle threshold (Ct) value below 25, which often coincides with the period of peak transmissibility [65]. On the regulatory side, the FDA granted Emergency Use Authorizations (EUAs) for multiple antigen tests during the pandemic and later advanced several to full approval [66]. For example, the BD Veritor™ System for SARS-CoV-2 antigen testing received full FDA approval in 2025, replacing its earlier EUA version. Multiplex antigen kits capable of detecting SARS-CoV-2 alongside influenza A and B have also been approved, further enhancing diagnostic efficiency [67].

### 4.3. Serological Testing

Serological testing detects pathogen-specific antibodies (e.g., IgM and IgG) produced after infection, providing a retrospective view of epidemic spread. However, it has limited value for early diagnosis [14]. During early COVID-19, IgM and IgG detection rates within the first two weeks after symptom onset were only about 50%. Reliable antibody detection generally requires several weeks [68]. Specificity is another challenge, as IgG results may be interfered by past infections or vaccination [69]. As such, serology is unsuitable for acute diagnosis but remains highly valuable for tracking epidemic and pandemic trends, assessing population immunity, and guiding public health policy.

Modern serological tests range from lateral flow immunoassays (LFAs) for point-of-care use to direct fluorescent antibody (DFA) assays, ELISAs, and chemiluminescent immunoassays (CLIAs) for high-throughput laboratory testing. Multiplex serological system represents a major advance. It can simultaneously detect antibodies against multiple viral proteins. This makes it possible to distinguish natural infection from vaccine-induced immunity [70]. For example, during the COVID-19 pandemic, an innovative serological test was developed that integrated a paper-based multiplex vertical flow assays (xVFAs) with a machine learning-based serodiagnostic algorithm. This system utilized a smartphone-based optical reader to capture assay signals and automatically classify individuals’ immunity status into three distinct categories: “protected,” “unprotected,” or “infected.” In a blind test, this integrated platform achieved an overall accuracy of 89.5%, demonstrating the potential of combining biosensing with AI for rapid immunity test [71].

Experience from COVID-19 also improved interpretation of combined diagnostic results. For example, “NAAT positive + antibody negative” suggests early infection, while “NAAT negative + antibody positive” may indicate recovery [72]. At the same time, antigenic cross-reactivity with other coronaviruses (e.g., 229E, OC43) can produce false positives, emphasizing the need for rigorous validation of test specificity [73]. In population seroprevalence studies, guidelines recommend prioritizing antibodies to the viral nucleocapsid (N) protein, rather than the spike (S) protein commonly targeted by vaccines, to avoid confounding effects of vaccination. These practices have helped standardize the clinical use of serology in epidemic and pandemic surveillance [74].

### 4.4. Next-Generation Sequencing (NGS)

Next-generation sequencing (NGS) has evolved from a research tool into an essential part of public health systems. Its primary value lies in viral genomic epidemiology [75]. Unlike targeted assays, NGS does not require prior knowledge of detection targets. It can sequence entire pathogen genomes, making it possible to clearly identify novel variants and important mutations. During the COVID-19 pandemic, the COVID-19 Genomics UK (COG-UK) Consortium established a distributed sequencing network that shared data in real time with global databases [76]. This provided an unprecedented view of transmission for variants of concern such as Delta and Omicron, offering critical information for public health decision-making [77].

In diagnostics, metagenomic NGS (mNGS) represents the leading edge of application. It enables high-throughput sequencing of all microorganisms, including viruses, bacteria, fungi, and parasites, directly from clinical samples. Studies have demonstrated that mNGS has significantly higher sensitivity than conventional microbiological methods. For example, in a multicenter retrospective study conducted in 2025, the detection rate of respiratory pathogens was 86.17% with mNGS, compared with 67.55% using traditional methods [78]. Optimized mNGS protocols have achieved an average detection limit as low as 543 copies/mL, with sensitivity and specificity both exceeding 93% [79]. Advanced bioinformatics pipelines, such as nf-UnO, designed specifically for outbreak investigation, can rapidly identify pathogens associated with epidemic and pandemic events [80]. These features make mNGS particularly valuable for outbreaks of unknown cause and position it as a powerful early warning tool for future “Disease X” scenarios.

### 4.5. Point-of-Care Testing (POCT)

POCT is best understood as a diagnostic strategy rather than a single technology. By integrating molecular methods, antigen testing, and microfluidics, it brings diagnostic capability directly to the site of patient care and supports rapid clinical decision-making [81]. POCT devices are portable, require minimal storage, produce results within 10–15 min, and can operate without specialized laboratory facilities. Many are CLIA-waived, making them suitable for use outside traditional laboratories [16]. However, POCT devices are often operated by non-professionals in uncontrolled environments, including for self-testing at home. These environments lack the standardized procedures and trained personnel of clinical laboratories, increasing the risk of pre-analytical errors (e.g., improper sample collection), analytical errors (e.g., incorrect timing or interpretation), and post-analytical errors (e.g., failure to report results), which sometimes lead to false positive results [51]. For this reason, widespread use must be accompanied by clear procedures and robust quality control. Key strategies include the following: First, utilizing digital technologies such as quick-response (QR) codes linked to video instructions, built-in timers, and smartphone applications that guide the user through the sampling process; Second, automatically transmitting POCT results to electronic health records or public health surveillance systems. This ensures data integrity, facilitates real-time monitoring; Third, sending known samples to testing sites (e.g., community clinics) for periodic proficiency testing to monitor performance.

Modern POCT platforms incorporate a variety of technologies, including LFAs, electrochemical biosensors, and miniaturized molecular diagnostic systems. Some devices can perform multiplex detection of several pathogens simultaneously. A major step forward has been smartphone-integrated POCT systems, which combine cloud connectivity for real-time data sharing with embedded machine learning algorithms for result interpretation [82]. These systems proved effective during the COVID-19 pandemic, facilitating large-scale testing while enhancing surveillance through remote data collection. Wearable biosensors represent another frontier in POCT. These devices continuously monitor physiological indicators such as heart rate, oxygen saturation, respiratory rate, and skin temperature. Studies during COVID-19 indicated that wearable platforms outperformed standard scoring systems such as NEWS2 in stratifying patients by viral load and predicting clinical deterioration. This capability supports 24-h remote monitoring, reduces direct patient, clinician contact, and lowers transmission risk [83]. Research is also advancing toward ultrasensitive biosensors. For example, work at the University of Florida is developing chip-based platforms designed to deliver results within seconds. Although most remain experimental, their potential speed and low-cost point to significant future gains in diagnostic capability [84].

**Figure 1:**
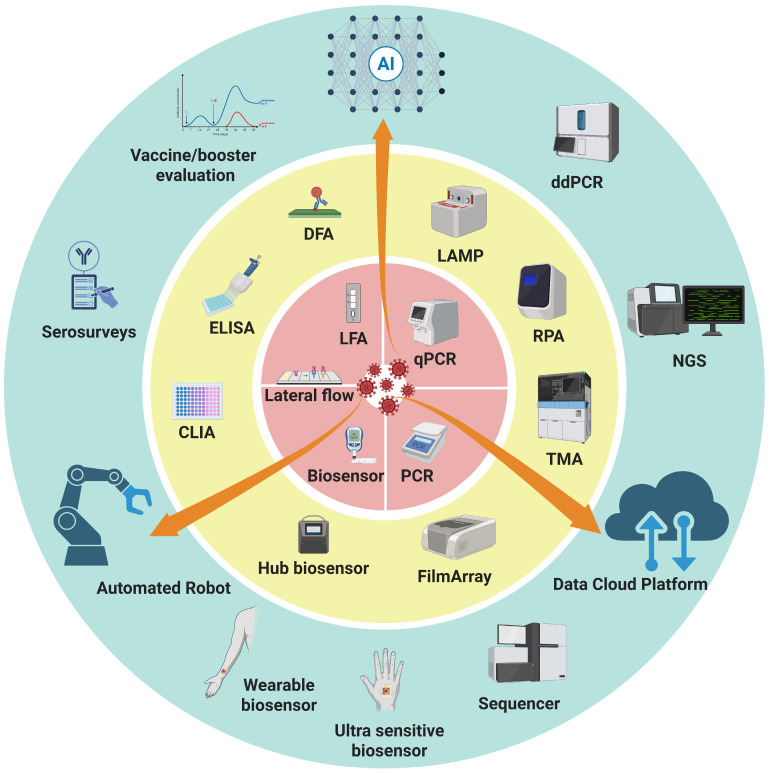
**Integrated framework of diagnostic technologies for epidemics and pandemics.** Three concentric rings of diagnostic strategies are presented: the inner ring (red) emphasizes speed and accessibility for timely, on-site detection, the middle ring (yellow) represents accuracy and throughput as the backbone of large-scale testing and case confirmation, and the outer ring (blue) reflects retrospective evaluation and advanced technologies that provide deeper insights for surveillance, variant tracking, and public health decision-making. Orange arrows spanning across the rings highlight the role of data-driven, automated solutions, and AI in connecting all layers, enabling real-time data sharing, automation of workflows, and intelligent interpretation. Abbreviations: AI, artificial intelligence; CLIA, chemiluminescent immunoassay; DFA, direct fluorescent antibody; ddPCR, droplet digital polymerase chain reaction; ELISA, enzyme-linked immunosorbent assay; LAMP, loop-mediated isothermal amplification; LFA, lateral flow assay; NGS, next-generation sequencing; POCT, point-of-care testing; qPCR, quantitative polymerase chain reaction; RPA, recombinase polymerase amplification; TMA, transcription-mediated amplification.

The WHO has proposed the “REASSURED” criteria for ideal rapid diagnostic tests: Real-time connectivity, Ease of specimen collection, Affordable, Sensitive, Specific, User-friendly, Rapid and robust, Equipment-free, and Deliverable to end users [85]. Future development of POCT must align with these principles, particularly by improving sensitivity, ensuring digital connectivity, and strengthening resilience against pathogen mutations [86]. Together, we integrated comparisons of performance, operational requirements, and key characteristics of these diagnostic technologies (Table 2). These advances show a multi-layered diagnostic system that connects laboratory testing with real-time response in the field (Figure 1).

### 4.6. Infectious Virus Quantification

One of the most significant gaps in current pandemic diagnostic capabilities is the accurate and rapid quantification of infectious viruses. While molecular diagnostics such as PCR and RT-PCR detect viral nucleic acids with high sensitivity and specificity, they cannot differentiate between infectious and non-infectious viral particles. This limitation has significant implications for clinical management and public health decision-making during epidemics and pandemics. Conventional approaches for infectious virus quantification rely primarily on cell culture-based methods, including plaque assays, focus-forming assays (FFA), and tissue culture infectious dose 50% (TCID50) assays. Plaque assays measure infectious viral particles by counting discrete plaques formed on susceptible cell monolayers, providing results in plaque-forming units (PFU) [87]. The FFA detect infected cells through immunofluorescence or enzymatic staining [88], and TCID50 method determines the viral dilution that infects 50% of cell cultures [89]. While these methods remain the gold standard for infectivity assessment, they have significant limitations. For example, their turnaround times typically range from 3–14 days, making them unsuitable for rapid clinical decision-making. The assays require specialized cell culture facilities, trained personnel, and strict biosafety containment, and are not amenable to rapid or high-throughput clinical reporting [90].

Recent technological advances have begun to address these limitations. Microfluidic digital focus assays represent a particularly promising development, it has both the specificity of focus-forming assays and the precision of digital quantification [91]. This technology combines a short-period cell culture (around 24 h) with a highly sensitive RNA detection step, such as RT-ddPCR, allows for the quantification of replication-competent virus by specifically detecting viral RNA amplification within infected cells, providing a result much faster than conventional culture while directly measuring infectivity [92]. Integration of microfluidics, digital quantification, and automated cell culture systems could potentially deliver infectious virus titers within 2–4 h. Other strategies include the use of reporter cell lines that express a detectable signal (e.g., fluorescence or luminescence) upon viral infection, enabling faster and more automated quantification of infectious units [93]. Additionally, some research focuses on correlating specific molecular signatures, such as the ratio of viral subgenomic to genomic RNA [94]. These technological advances would transform clinical practice, enabling same-day decisions about patient isolation, or transmission risk assessment.

## 5. Future Goals for Epidemic and Pandemic Diagnostics

Innovations in laboratory diagnostics over the past two decades have significantly transformed the landscape of epidemic and pandemic response. Among these innovations, the acceleration of turnaround times for patient sampling and diagnostic results stands out as a critical factor in improving clinical outcomes and public health effectiveness. The deployment of rapid, high throughput molecular technologies, allowing results within 15–90 min, ddPCR and multiplex platforms further enable high-throughput sample processing. POCT, enhanced by miniaturization, biosensing, and AI, has moved diagnosis to the patient bedside, community clinics, and even self-testing at home. Simultaneously, distributed screening and real-time data sharing facilitate faster public health interventions, including contact tracing and community containment measures, effectively shortening the overall response cycle. These advancements have collectively built a solid technological foundation for our capacity to respond to epidemics and pandemics.

The lessons of pandemics, particularly COVID-19, have prompted the international community to re-examine epidemic preparedness and design a blueprint for the future. At the core of this blueprint is the integration of technological innovation with global collaboration, ensuring that scientific advances can be rapidly translated into effective public health action during the next crisis. In recognition of this need, WHO member states adopted the historic Pandemic Agreement in 2025. This agreement aims to build a stronger and fairer global health system by ensuring equitable access to diagnostics tools and promoting “whole-of-government, whole-of-society” participation [95]. To succeed, the accord must be supported by practical collaboration. Global initiatives such as the “100 Days Mission” have set ambitious goals, targeting the availability of countermeasures within 100 days of identifying a new epidemic threat. Meeting this target requires the development and pre-positioning of rapid diagnostic tools and highlights the urgency of investing in research on high-priority pathogens during inter-epidemic periods [96]. In addition, regulatory pathways for infectious virus quantification assays remain poorly defined. Establishing standardized protocols, reference materials, and performance criteria for infectious virus quantification will be essential for widespread adoption and regulatory approval [97]. International harmonization of these standards could facilitate rapid deployment during future pandemic responses.

The laboratory medicine community has a pivotal role to play. Collaboration should focus on building sustainable capacity in low- and middle-income countries, where shortages of laboratory professionals and infrastructure remain critical barriers. Long-term solutions may include laboratory twinning programs, virtual training networks, and standardized quality systems to strengthen local diagnostic capacity and ensure resilience against future outbreaks [98]. Ultimately, the effectiveness of all technical advances and cooperative strategies depends on adopting a broader guiding principle: the One Health framework. This approach emphasizes interdisciplinary collaboration across human, animal, and environmental health. Because most emerging infectious diseases originate from zoonoses, integrating veterinary surveillance, environmental monitoring, and human health data is essential to provide early warning of pathogen and detect pandemics at their source [99].

In summary, the future of epidemic and pandemic diagnostics and control requires a systematic approach that unites technology, policy, and governance. By advancing diagnostic innovation, upholding the equity principles of the Pandemic Accord, and implementing the collaborative vision of One Health, the global community can build a stronger health defense system and reduce the destructive impact of future crises.

## Figures and Tables

**Table 1:** Comparison of diagnostic challenges, advances, and lessons learned across three major epidemic and pandemic events.

	Diagnostic Challenges	Technological Advances	Lessons Learned
SARS (2002–2003)	Pathogen initially unknown Low viral load in early stage	Enabled PCR development Increased investment in molecular diagnostics	Real-time interconnected data systems are needed Molecular diagnostic is fundamental
H1N1pdm09 (2009–2010)	Antigen test sensitivity limitations Laboratory capacity overwhelmed Inadequate global surveillance infrastructure	RT-PCR assay development and deployment Expansion in molecular testing capacity	Importance of molecular diagnostics Global surveillance network strengthening essential
Ebola (2014–2016)	Local diagnostic capacity absent Samples shipped abroad delays Cold-chain transport difficult Blood collection risked infection	Mobile laboratories enabled frontline testing Onsite rapid tests reduce turnaround time	Frontline diagnostic capacity is essential Value of distributed diagnostic models
COVID-19 (2019–2023)	Major insufficient and inequality in global testing capacity Global supply chain shortages	Ultra–large-scale capacity via automation Widespread rapid antigen testing, self-testing NGS and Intelligent POCT	Multi-level and complementary diagnostic system Resilient supply chains and regulatory Global cooperation and data sharing are essential

**Table 2:** Comprehensive comparison of major diagnostic technologies for pandemic response.

Technology	Limit of Detection (LoD)	Sensitivity/Specificity	Approx. Turnaround Time (TAT)	Cost per Test	Throughput	Technical Complexity & Infrastructure	Vulnerability to Variants	Typical Regulatory Status
PCR/RT-PCR	10^2^–10^3^ copies/mL	High (>95%)/High (>95%)	60–120 min (after extraction)	$10–$15	Medium to High (with automation)	High (thermal cycler, lab, trained staff)	Low (if target conserved)	FDA-Cleared, CE-Marked, WHO EUL
LAMP	10^2^–10^4^ copies/mL	Comparable to PCR/High	30–60 min	<$10	Medium to High (platform-dependent)	Low to Medium (isothermal block, simple reader)	Low (if target conserved)	Increasing EUAs/Approvals
RPA	10^2^–10^3^ copies/mL	High/High (platform-dependent)	10–20 min	<$10	Low	Very Low (isothermal, portable)	Low (if target conserved)	Emerging, some EUAs
TMA	10^1^–10^2^ copies/mL	Very High/Very High	30–60 min	$10–$15	High (integrated automated systems)	Medium (dedicated automated instrument)	Low (if target conserved)	FDA-Cleared (e.g., blood screening)
ddPCR	1–10 copies per reaction	Very High/Very High	180–240 min	>$150	Low to Medium	High (droplet generator, reader, expert analysis)	Low (if target conserved)	Typical LDT-based
Antigen Test	10^4^–10^6^ copies/mL	Moderate-High/High (varies with viral load)	15–30 min	<$10	Low to Medium	Very Low (visual read, no instrument)	High (if target epitope mutates)	FDA EUA/Approval, WHO EUL, CE-Marked
Serology (IgG/IgM)	N/A (qualitative)	Moderate-High/Moderate-High (varies by assay)	15 min–2 h	<$10	Low to High (platform-dependent)	Low (LFA) to High (CLIA/ELISA)	Low to Moderate (if antigenic drift)	FDA EUA/Approval, CE-Marked
NGS/mNGS	Targeted NGS 10^2^–10^4^ mNGS 10^3^–10^5^	Very High/High (bioinformatics-dependent)	24–72 h	>$150	Low (per sample)	Very High (sequencer, computing, bioinformaticians)	None (agnostic)	Typical LDT-based
POCT	NAAT-POCT 10^2^–10^4^ antigen-POCT 10^4^–10^6^	High/High	20–45 min	$10–$15	Low	Low (all-in-one cartridge system)	Low (if target conserved)	FDA-Cleared (CLIA-waived)

Abbreviations: CE, conformité européenne (european conformity); CLIA, clinical laboratory improvement amendments; EUA, emergency use authorization; EUL, emergency use listing; FDA, U.S. food and drug administration; IVD: in vitro diagnostic; LDT, laboratory developed tests; WHO, world health organization.

## Data Availability

The original contributions presented in this study are included in the article. Further inquiries can be directed to the corresponding author.

## References

[1] Wang S., Li W., Wang Z., Yang W., Li E., Xia X., Yan F., Chiu S. (2024). Emerging and reemerging infectious diseases: Global trends and new strategies for their prevention and control. Signal Transduct. Target. Ther..

[2] Matthews Q., da Silva S.J.R., Norouzi M., Pena L.J., Pardee K. (2020). Adaptive, diverse and de-centralized diagnostics are key to the future of outbreak response. BMC Biol..

[3] (2003). WHO Disease Outbreak Reported. https://www.who.int/emergencies/disease-outbreak-news/item/2003_04_11-en.

[4] Briand S., Mounts A., Chamberland M. (2011). Challenges of global surveillance during an influenza pandemic. Public Health.

[5] Ellerbrok H., Jacobsen S., Patel P., Rieger T., Eickmann M., Becker S., Gunther S., Naidoo D., Schrick L., Keeren K. (2017). External quality assessment study for ebolavirus PCR-diagnostic promotes international preparedness during the 2014–2016 Ebola outbreak in West Africa. PLoS Neglected Trop. Dis..

[6] Das S., Dunbar S. (2022). The COVID-19 Pandemic—A Diagnostic Industry Perspective. Front. Cell. Infect. Microbiol..

[7] (2024). U.S. Government Global Health Security Strategy. https://bidenwhitehouse.archives.gov/wp-content/uploads/2024/04/Global-Health-Security-Strategy-2024-1.pdf.

[8] Monis P.T., Giglio S. (2006). Nucleic acid amplification-based techniques for pathogen detection and identification. Infect. Genet. Evol..

[9] Srivastava P., Prasad D. (2023). Isothermal nucleic acid amplification and its uses in modern diagnostic technologies. 3 Biotech.

[10] Pham J., Meyer S., Nguyen C., Williams A., Hunsicker M., McHardy I., Gendlina I., Goldstein D.Y., Fox A.S., Hudson A. (2020). Performance Characteristics of a High-Throughput Automated Transcription-Mediated Amplification Test for SARS-CoV-2 Detection. J. Clin. Microbiol..

[11] Lobato I.M., O’Sullivan C.K. (2018). Recombinase polymerase amplification: Basics, applications and recent advances. TrAC Trends Anal. Chem..

[12] Li H., Bai R., Zhao Z., Tao L., Ma M., Ji Z., Jian M., Ding Z., Dai X., Bao F. (2018). Application of droplet digital PCR to detect the pathogens of infectious diseases. Biosci. Rep..

[13] Smith D.R.M., Duval A., Zahar J.R., Opatowski L., Temime L., Hendrickx N., Jean K., Jijón S., Oodally A., Shirreff G. (2022). Rapid antigen testing as a reactive response to surges in nosocomial SARS-CoV-2 outbreak risk. Nat. Commun..

[14] Hayden M.K., El Mikati I.K., Hanson K.E., Englund J.A., Humphries R.M., Lee F., Loeb M., Morgan D.J., Patel R., Al Ta’ani O. (2024). Infectious Diseases Society of America Guidelines on the Diagnosis of COVID-19: Serologic Testing. Clin. Infect. Dis..

[15] Gwinn M., MacCannell D., Armstrong G.L. (2019). Next-Generation Sequencing of Infectious Pathogens. JAMA.

[16] Yang S.M., Lv S., Zhang W., Cui Y. (2022). Microfluidic Point-of-Care (POC) Devices in Early Diagnosis: A Review of Opportunities and Challenges. Sensors.

[17] Cherry J.D. (2004). The chronology of the 2002–2003 SARS mini pandemic. Paediatr. Respir. Rev..

[18] Berger A., Drosten C., Doerr H.W., Sturmer M., Preiser W. (2004). Severe acute respiratory syndrome (SARS)—Paradigm of an emerging viral infection. J. Clin. Virol..

[19] Chan P.K., To W.K., Ng K.C., Lam R.K., Ng T.K., Chan R.C., Wu A., Yu W.C., Lee N., Hui D.S. (2004). Laboratory diagnosis of SARS. Emerg. Infect. Dis..

[20] Goddard N.L., Delpech V.C., Watson J.M., Regan M., Nicoll A. (2006). Lessons learned from SARS: The experience of the Health Protection Agency, England. Public Health.

[21] Heymann D.L., Rodier G. (2004). Global surveillance, national surveillance, and SARS. Emerg. Infect. Dis..

[22] Grein T., Leitmeyer K., Mardel S., Merianos A., Olowokure B., Roth C., Slattery R. (2004). The WHO response to SARS and preparations for the future. Learning from SARS: Preparing for the Next Disease Outbreak: Workshop Summary.

[23] Shrestha S.S., Swerdlow D.L., Borse R.H., Prabhu V.S., Finelli L., Atkins C.Y., Owusu-Edusei K., Bell B., Mead P.S., Biggerstaff M. (2011). Estimating the burden of 2009 pandemic influenza A (H1N1) in the United States (April 2009–April 2010). Clin. Infect. Dis..

[24] Hayden R.T., Wick M.T., Rodriguez A.B., Caliendo A.M., Mitchell M.J., Ginocchio C.C. (2010). A survey-based assessment of United States clinical laboratory response to the 2009 H1N1 influenza outbreak. Arch. Pathol. Lab. Med..

[25] Polansky L.S., Outin-Blenman S., Moen A.C. (2016). Improved global capacity for influenza surveillance. Emerg. Infect. Dis..

[26] (2014). Updated Preparedness and Response Framework for Influenza Pandemics. https://www.cdc.gov/mmwr/preview/mmwrhtml/rr6306a1.htm.

[27] (2017). Guide to Revision of National Pandemic Influenza Preparedness Plans. https://www.ecdc.europa.eu/sites/default/files/documents/Guide-to-pandemic-preparedness-revised.pdf.

[28] Parada L.V. (2011). Public health: Life lessons. Nature.

[29] Frieden T.R., Damon I., Bell B.P., Kenyon T., Nichol S. (2014). Ebola 2014—New challenges, new global response and responsibility. N. Engl. J. Med..

[30] Kyobe Bosa H., Kamara N., Aragaw M., Wayengera M., Talisuna A., Bangura J., Mwebesa H.G., Katoto P., Agyarko R.K., Ihekweazu C. (2024). The west Africa Ebola virus disease outbreak: 10 years on. Lancet Glob. Health.

[31] McNamara L.A. (2016). Ebola Surveillance—Guinea, Liberia, and Sierra Leone. MMWR Suppl..

[32] Wang L., Brima Tia A., Xu B., Qi X., Harding D. (2023). Sustainable Laboratory Capacity Building in Sierra Leone: From Ebola to COVID-19. China CDC Wkly..

[33] Kadanali A., Karagoz G. (2015). An overview of Ebola virus disease. North. Clin. Istanb..

[34] Presser L.D., Coffin J., Koivogui L., Campbell A., Campbell J., Barrie F., Ngobeh J., Souma Z., Sorie S., Harding D. (2021). The deployment of mobile diagnostic laboratories for Ebola virus disease diagnostics in Sierra Leone and Guinea. Afr. J. Lab. Med..

[35] Semper A.E., Broadhurst M.J., Richards J., Foster G.M., Simpson A.J., Logue C.H., Kelly J.D., Miller A., Brooks T.J., Murray M. (2016). Performance of the GeneXpert Ebola Assay for Diagnosis of Ebola Virus Disease in Sierra Leone: A Field Evaluation Study. PLoS Med..

[36] Katawera V., Kohar H., Mahmoud N., Raftery P., Wasunna C., Humrighouse B., Hardy P., Saindon J., Schoepp R., Makvandi M. (2019). Enhancing laboratory capacity during Ebola virus disease (EVD) heightened surveillance in Liberia: Lessons learned and recommendations. Pan Afr. Med. J..

[37] Haakenstad A., Irvine C.M.S., Knight M., Bintz C., Aravkin A.Y., Zheng P., Gupta V., Abrigo M.R., Abushouk A.I., Adebayo O.M.J.T.L. (2022). Measuring the availability of human resources for health and its relationship to universal health coverage for 204 countries and territories from 1990 to 2019: A systematic analysis for the Global Burden of Disease Study 2019. Lancet.

[38] Bektemur G., Muzoglu N., Arici M.A., Karaaslan M.K. (2018). Cost analysis of medical device spare parts. Pak. J. Med. Sci..

[39] Yadav H., Shah D., Sayed S., Horton S., Schroeder L.F. (2021). Availability of essential diagnostics in ten low-income and middle-income countries: Results from national health facility surveys. Lancet Glob. Health.

[40] Gwaza G., Pluddemann A., McCall M., Heneghan C. (2024). Integrated Diagnosis in Africa’s Low- and Middle-Income Countries: What Is It, What Works, and for Whom? A Realist Synthesis. Int. J. Integr. Care.

[41] Espinal M.A., Salomon M.L., Periago M.R. (2025). PAHO is Latin America’s Centre for Disease Control and Prevention. Lancet.

[42] Al-Waleedi A.A., Thabet A.A., Bin Azoon N., Dandarwe A., Al-Amoudi A.S., Al-Gailani A., Atef B. (2023). An assessment of the current epidemiological and laboratory capacities for influenza-like illnesses and severe acute respiratory infection surveillance, Yemen 2022. Influenza Other Respir Viruses.

[43] Shultz J.M., Perlin A., Saltzman R.G., Espinel Z., Galea S. (2020). Pandemic March: 2019 Coronavirus Disease’s First Wave Circumnavigates the Globe. Disaster Med. Public Health Prep..

[44] (2023). WHO COVID-19 Dashboard. https://data.who.int/dashboards/covid19/cases?n=c.

[45] (2024). COVID-19 Eliminated a Decade of Progress in Global Level of Life Expectancy. https://www.who.int/news/item/24-05-2024-covid-19-eliminated-a-decade-of-progress-in-global-level-of-life-expectancy.

[46] Aguiar E., Navas J., Pacheco L.G.C. (2020). The COVID-19 Diagnostic Technology Landscape: Efficient Data Sharing Drives Diagnostic Development. Front. Public Health.

[47] Das S., Frank K.M. (2022). Strategies for Scaling up SARS-CoV-2 Molecular Testing Capacity. Clin. Lab. Med..

[48] Rosella L.C., Agrawal A., Gans J., Goldfarb A., Sennik S., Stein J. (2022). Large-scale implementation of rapid antigen testing system for COVID-19 in workplaces. Sci. Adv..

[49] Wang W., Xu Y., Gao R., Lu R., Han K., Wu G., Tan W. (2020). Detection of SARS-CoV-2 in Different Types of Clinical Specimens. JAMA.

[50] Hung I.F., Cheng V.C., Li X., Tam A.R., Hung D.L., Chiu K.H., Yip C.C., Cai J.P., Ho D.T., Wong S.C. (2020). SARS-CoV-2 shedding and seroconversion among passengers quarantined after disembarking a cruise ship: A case series. Lancet Infect. Dis..

[51] Fang B., Meng Q.H. (2020). The laboratory’s role in combating COVID-19. Crit. Rev. Clin. Lab. Sci..

[52] Martinez M.J., Cotten M., Phan M.V.T., Becker K., Espasa M., Leegaard T.M., Lisby G., Schneider U.V., Casals-Pascual C. (2024). Viral epidemic preparedness: A perspective from five clinical microbiology laboratories in Europe. Clin. Microbiol. Infect..

[53] Mercer T.R., Salit M. (2021). Testing at scale during the COVID-19 pandemic. Nat. Rev. Genet..

[54] Walter J.M., Wunderink R.G. (2017). Severe Respiratory Viral Infections: New Evidence and Changing Paradigms. Infect. Dis. Clin. North. Am..

[55] van Beuningen R., Jim K.K., Boot M., Ossendrijver M., Keijser B.J., van de Bovenkamp J.H., Melchers W.J., Kievits T. (2024). Development of a large-scale rapid LAMP diagnostic testing platform for pandemic preparedness and outbreak response. Biol. Methods Protoc..

[56] Moron-Lopez S., Riveira-Munoz E., Urrea V., Gutierrez-Chamorro L., Avila-Nieto C., Noguera-Julian M., Carrillo J., Mitja O., Mateu L., Massanella M. (2023). Comparison of Reverse Transcription (RT)-Quantitative PCR and RT-Droplet Digital PCR for Detection of Genomic and Subgenomic SARS-CoV-2 RNA. Microbiol. Spectr..

[57] Kinloch N.N., Ritchie G., Dong W., Cobarrubias K.D., Sudderuddin H., Lawson T., Matic N., Montaner J.S.G., Leung V., Romney M.G. (2021). SARS-CoV-2 RNA Quantification Using Droplet Digital RT-PCR. J. Mol. Diagn..

[58] Secchi V., Armanni A., Barbieri L., Bruno A., Colombo A., Fumagalli S., Kukushkina E.A., Lorenzi R., Marchesi L., Moukham H. (2025). Advanced techniques and nanotechnologies for point-of-care testing. Front. Nanotechnol..

[59] Taylor S.C., Laperriere G., Germain H. (2017). Droplet Digital PCR versus qPCR for gene expression analysis with low abundant targets: From variable nonsense to publication quality data. Sci. Rep..

[60] Hanson K.E., Azar M.M., Banerjee R., Chou A., Colgrove R.C., Ginocchio C.C., Hayden M.K., Holodiny M., Jain S., Koo S. (2020). Molecular Testing for Acute Respiratory Tract Infections: Clinical and Diagnostic Recommendations From the IDSA’s Diagnostics Committee. Clin. Infect. Dis..

[61] (2025). Seegene Unveils CURECA™ and STAgora™ at ADLM. https://www.seegene.com/press_release/seegene_unveils_cureca_and_stagora_at_adlm_2025_advancing_the_next_stage_of_diagnostics__2025.

[62] Azar M.M., Landry M.L. (2018). Detection of Influenza A and B Viruses and Respiratory Syncytial Virus by Use of Clinical Laboratory Improvement Amendments of 1988 (CLIA)-Waived Point-of-Care Assays: A Paradigm Shift to Molecular Tests. J. Clin. Microbiol..

[63] Ford L., Lee C., Pray I.W., Cole D., Bigouette J.P., Abedi G.R., Bushman D., Delahoy M.J., Currie D.W., Cherney B. (2021). Epidemiologic Characteristics Associated With Severe Acute Respiratory Syndrome Coronavirus 2 (SARS-CoV-2) Antigen-Based Test Results, Real-Time Reverse Transcription Polymerase Chain Reaction (rRT-PCR) Cycle Threshold Values, Subgenomic RNA, and Viral Culture Results From University Testing. Clin. Infect. Dis..

[64] Barlev-Gross M., Weiss S., Ben-Shmuel A., Sittner A., Eden K., Mazuz N., Glinert I., Bar-David E., Puni R., Amit S. (2021). Spike vs nucleocapsid SARS-CoV-2 antigen detection: Application in nasopharyngeal swab specimens. Anal. Bioanal. Chem..

[65] Arumugam S., Ma J., Macar U., Han G., McAulay K., Ingram D., Ying A., Chellani H.H., Chern T., Reilly K. (2023). Rapidly adaptable automated interpretation of point-of-care COVID-19 diagnostics. Commun. Med..

[66] (2023). In Vitro Diagnostics EUAs. https://www.fda.gov/medical-devices/covid-19-emergency-use-authorizations-medical-devices/in-vitro-diagnostics-euas-antigen-diagnostic-tests-sars-cov-2.

[67] Kilic A., Hiestand B., Palavecino E. (2021). Evaluation of Performance of the BD Veritor SARS-CoV-2 Chromatographic Immunoassay Test in Patients with Symptoms of COVID-19. J. Clin. Microbiol..

[68] Deeks J.J., Dinnes J., Takwoingi Y., Davenport C., Spijker R., Taylor-Phillips S., Adriano A., Beese S., Dretzke J., Ferrante di Ruffano L. (2020). Antibody tests for identification of current and past infection with SARS-CoV-2. Cochrane Database Syst. Rev..

[69] Talbot H.K., Falsey A.R. (2010). The diagnosis of viral respiratory disease in older adults. Clin. Infect. Dis..

[70] Stern D., Meyer T.C., Treindl F., Mages H.W., Krueger M., Skiba M., Krueger J.P., Zobel C.M., Schreiner M., Grossegesse M.J.S.R. (2023). A bead-based multiplex assay covering all coronaviruses pathogenic for humans for sensitive and specific surveillance of SARS-CoV-2 humoral immunity. Sci. Rep..

[71] Eryilmaz M., Goncharov A., Han G.R., Joung H.A., Ballard Z.S., Ghosh R., Zhang Y., Di Carlo D., Ozcan A. (2024). A Paper-Based Multiplexed Serological Test to Monitor Immunity against SARS-COV-2 Using Machine Learning. ACS Nano.

[72] Harvey R.A., Rassen J.A., Kabelac C.A., Turenne W., Leonard S., Klesh R., Meyer W.A., Kaufman H.W., Anderson S., Cohen O. (2021). Association of SARS-CoV-2 Seropositive Antibody Test With Risk of Future Infection. JAMA Intern. Med..

[73] Nordgren J., Sharma S., Olsson H., Jamtberg M., Falkeborn T., Svensson L., Hagbom M. (2021). SARS-CoV-2 rapid antigen test: High sensitivity to detect infectious virus. J. Clin. Virol..

[74] (2019). Interim Guidelines for COVID-19 Antibody Testing. https://archive.cdc.gov/www_cdc_gov/coronavirus/2019-ncov/hcp/testing/antibody-tests-guidelines.html.

[75] Gu W., Miller S., Chiu C.Y. (2019). Clinical Metagenomic Next-Generation Sequencing for Pathogen Detection. Annu. Rev. Pathol..

[76] Marjanovic S., Romanelli R.J., Ali G.C., Leach B., Bonsu M., Rodriguez-Rincon D., Ling T. (2022). COVID-19 Genomics UK (COG-UK) Consortium: Final Report. Rand Health Q..

[77] McCrone J.T., Hill V., Bajaj S., Pena R.E., Lambert B.C., Inward R., Bhatt S., Volz E., Ruis C., Dellicour S. (2022). Context-specific emergence and growth of the SARS-CoV-2 Delta variant. Nature.

[78] Chen S., Ouyang T., Wang K., Hou X., Zhang R., Li M., Zhang H., He Q., Li X., Liu Z. (2025). Application of metagenomic next-generation sequencing in pathogen detection of lung infections. Front. Cell. Infect. Microbiol..

[79] Tan J.K., Servellita V., Stryke D., Kelly E., Streithorst J., Sumimoto N., Foresythe A., Huh H.J., Nguyen J., Oseguera M.J.N.c. (2024). Laboratory validation of a clinical metagenomic next-generation sequencing assay for respiratory virus detection and discovery. Nat. Commun..

[80] Guzman-Cole C., Huang A.D. (2025). nf-UnO pipeline: A metagenomic co-assembly pipeline for novel pathogen detection from mNGS outbreak sets. Bioinformatics.

[81] Sachdeva S., Davis R.W., Saha A.K. (2020). Microfluidic Point-of-Care Testing: Commercial Landscape and Future Directions. Front. Bioeng. Biotechnol..

[82] Zayed B.A., Ali A.N., Elgebaly A.A., Talaia N.M., Hamed M., Mansour F.R. (2023). Smartphone-based point-of-care testing of the SARS-CoV-2: A systematic review. Sci. Afr..

[83] Un K.C., Wong C.K., Lau Y.M., Lee J.C., Tam F.C., Lai W.H., Lau Y.M., Chen H., Wibowo S., Zhang X. (2021). Observational study on wearable biosensors and machine learning-based remote monitoring of COVID-19 patients. Sci. Rep..

[84] Li L., Song M., Lao X., Pang S.-Y., Liu Y., Wong M.-C., Ma Y., Yang M., Hao J. (2022). Rapid and ultrasensitive detection of SARS-CoV-2 spike protein based on upconversion luminescence biosensor for COVID-19 point-of-care diagnostics. Mater. Des..

[85] Land K.J., Boeras D.I., Chen X.S., Ramsay A.R., Peeling R.W. (2019). REASSURED diagnostics to inform disease control strategies, strengthen health systems and improve patient outcomes. Nat. Microbiol..

[86] Yimer S.A., Booij B.B., Tobert G., Hebbeler A., Oloo P., Brangel P., L’Azou Jackson M., Jarman R., Craig D., Avumegah M.S. (2024). Rapid diagnostic test: A critical need for outbreak preparedness and response for high priority pathogens. BMJ Glob. Health.

[87] Baer A., Kehn-Hall K. (2014). Viral concentration determination through plaque assays: Using traditional and novel overlay systems. J. Vis. Exp..

[88] Battegay M., Cooper S., Althage A., Bänziger J., Hengartner H., Zinkernagel R.M. (1991). Quantification of lymphocytic choriomeningitis virus with an immunological focus assay in 24-or 96-well plates. J. Virol. Methods.

[89] LaBarre D.D., Lowy R.J. (2001). Improvements in methods for calculating virus titer estimates from TCID50 and plaque assays. J. Virol. Methods.

[90] Pastorino B., Touret F., Gilles M., Luciani L., de Lamballerie X., Charrel R.N. (2020). Evaluation of Chemical Protocols for Inactivating SARS-CoV-2 Infectious Samples. Viruses.

[91] Vafadar A., Takallu S., Alashti S.K., Rashidi S., Bahrani S., Tajbakhsh A., Mirzaei E., Savardashtaki A. (2024). Advancements in microfluidic platforms for rapid biomarker diagnostics of infectious diseases. Microchem. J..

[92] Srimathi S.R., Ignacio M.A., Rife M., Tai S., Milton D.K., Scull M.A., DeVoe D.L. (2025). Microfluidic digital focus assays for the quantification of infectious influenza virus. Lab Chip.

[93] Desmarets L., Callens N., Hoffmann E., Danneels A., Lavie M., Couturier C., Dubuisson J., Belouzard S., Rouille Y. (2022). A reporter cell line for the automated quantification of SARS-CoV-2 infection in living cells. Front. Microbiol..

[94] van Kampen J.J.A., van de Vijver D., Fraaij P.L.A., Haagmans B.L., Lamers M.M., Okba N., van den Akker J.P.C., Endeman H., Gommers D., Cornelissen J.J. (2021). Duration and key determinants of infectious virus shedding in hospitalized patients with coronavirus disease-2019 (COVID-19). Nat. Commun..

[95] Parums D.V. (2025). Editorial: The 2025 World Health Assembly Pandemic Agreement and the 2024 Amendments to the International Health Regulations Combine for Pandemic Preparedness and Response. Med. Sci. Monit..

[96] Barnsley G., Mesa D.O., Hogan A.B., Winskill P., A Torkelson A., Walker D.G., Ghani A.C., Watson O.J. (2024). Impact of the 100 days mission for vaccines on COVID-19: A mathematical modelling study. Lancet Glob. Health.

[97] Agard A., Elsheikh O., Bell D., Relich R.F., Schmitt B.H., Sadowski J., Fadel W., Webb D.H., Dbeibo L., Kelley K. (2022). Clinical comparison and agreement of PCR, antigen, and viral culture for the diagnosis of COVID-19: Clinical Agreement Between Diagnostics for COVID19. J. Clin. Virol. Plus.

[98] Nkengasong J.N., Yao K., Onyebujoh P. (2018). Laboratory medicine in low-income and middle-income countries: Progress and challenges. Lancet.

[99] Okesanya O.J., Olatunji G., Manirambona E., Oluebube M.M., Rasheed A.A., Olaleke N.O., Ogunlayi A.C., Ogaya J.B., Oladipo E.K., Igbalajobi O.A. (2023). Synergistic fight against future pandemics: Lessons from previous pandemics. Infez. Med..

